# A Computational Study of the Shear Behavior of Reinforced Concrete Beams Affected from Alkali–Silica Reactivity Damage

**DOI:** 10.3390/ma14123346

**Published:** 2021-06-17

**Authors:** Bora Gencturk, Hadi Aryan, Mohammad Hanifehzadeh, Clotilde Chambreuil, Jianqiang Wei

**Affiliations:** 1 Sonny Astani Department of Civil and Environmental Engineering, University of Southern California, 3620 S. Vermont Avenue, KAP 210, Los Angeles, CA 90089-2531, USA; haryan@usc.edu (H.A.); hanifzadehm@gmail.com (M.H.); 2 LMT—Laboratoire de Mécanique et Technologie, University Paris-Saclay, ENS Paris-Saclay, CNRS, 91190 Gif-sur-Yvette, France; clotilde.chambreuil@ens-paris-saclay.fr; 3 Department of Civil and Environmental Engineering, University of Massachusetts, Lowell, MA 01854-5104, USA; Jianqiang_Wei@uml.edu

**Keywords:** reinforced concrete, shear failure, alkali–silica reactivity, finite element modeling

## Abstract

In this study, an investigation of the shear behavior of full-scale reinforced concrete (RC) beams affected from alkali–silica reactivity damage is presented. A detailed finite element model (FEM) was developed and validated with data obtained from the experiments using several metrics, including a force–deformation curve, rebar strains, and crack maps and width. The validated FEM was used in a parametric study to investigate the potential impact of alkali–silica reactivity (ASR) degradation on the shear capacity of the beam. Degradations of concrete mechanical properties were correlated with ASR expansion using material test data and implemented in the FEM for different expansions. The finite element (FE) analysis provided a better understanding of the failure mechanism of ASR-affected RC beam and degradation in the capacity as a function of the ASR expansion. The parametric study using the FEM showed 6%, 19%, and 25% reduction in the shear capacity of the beam, respectively, affected from 0.2%, 0.4%, and 0.6% of ASR-induced expansion.

## 1. Introduction

The durability of concrete is an important consideration for reinforced concrete (RC) structures. One of the major concerns is the degradation of concrete from alkali–silica reaction (ASR), which is a slowly occurring chemical process between the hydroxyl ions available in the pore water of concrete and the aggregate containing reactive silica. The chemical product, named ASR gel, is highly expansive in the presence of moisture. The gel expansion develops an internal pressure and induces micro-cracking, which degrades the mechanical and physical properties of the concrete [1]. As micro-cracks build up throughout the concrete volume, the negative effects of the ASR on the macro-scale properties of concrete are observed. Substantial damage in the form of both micro and macrocracking has been reported in various types of structures in the United States, including bridges, roadways, dams and nuclear power plants due to ASR [2,3,4]. The initial sign of ASR is mapping cracks on the exposed concrete surfaces. Mapping cracks are a pattern of hairline cracks with random orientation on the surface of a concrete member. Over time, additional symptoms, including volumetric expansion, relative movement, localized crushing and spalling, and surface discoloration may be observed. In extreme cases, the rupture of the reinforcement due to excessive expansion strains has been reported [5]. The rate of expansion is largely a function of the reactivity of the aggregate, the alkali content of the cement, the confinement level of the structural element, and the environmental conditions, i.e., moisture and temperature [6,7]. In some cases, up to 0.6% expansion was observed in a period of 10 years in the field [7]. The expansion and cracking eventually result in a reduction in the service life of the structure. In addition to the field evidence and experimental studies, numerical models have been developed to simulate the concrete expansion and deterioration due to ASR. Recently, ASR degradation has been implemented in lattice discrete particle models (LDPM), which can simulate the heterogenous nature of concrete at the coarse aggregate scale. This approach enables an accurate modeling of the volumetric expansion of the ASR gel, concrete cracking and the degradation of concrete strength and stiffness due to ASR [8,9,10].

The shear failure of RC beams is relatively less understood; particularly, for damaged infrastructure components. Sucharda [11] presented a procedure to identify the fracture properties of concrete through inverse analysis of multicriteria decision analysis, stochastic modelling and nonlinear simulations. The identified parameters were then used to perform a parametric study on the shear behavior of concrete beams with no shear reinforcement. The basic mechanical properties of concrete were defined through laboratory tests and a number of concrete beams with different longitudinal reinforcement ratios were tested in shear. In addition to determining the fracture energy, the shear capacity as a function of longitudinal reinforcement ratio was derived. Conforti and Minelli [12] performed a numerical study on the shear behavior of fiber-reinforced concrete deep beams using the modified compression field theory and disturbed stress field model. Particular attention was given to the modeling of nonlinear properties of concrete such as tension softening. After validating the models with experimental results, the influence of size effect on the shear behavior of the beams was investigated. Nine beams without shear reinforcement were studied in three groups of no fiber, and 50 kg/m^3^ and 75 kg/m^3^ of hooked-end steel fibers. All beams were 250 mm wide, and each group included three beams each with 500 mm, 1000 mm, and 1500 mm height and an effective depth 60 mm less than the beam height. The shear span for each beam was three times the effective depth of the beam. The results showed that the modified compression field theory and disturbed stress field model accurately predict the shear behavior of different size beams with and without fibers. Wu and Hu [13] presented an experimental method to quantify the contributions of concrete and transverse reinforcement to the shear strength of RC beams. The developed approach was based on measuring the strains along the entire length of each stirrup without affecting the bond between the reinforcement and the concrete. It was concluded that not all the stirrups intersecting a major diagonal shear crack yield at the verge of shear strength. Additionally, the peak contributions of concrete and transverse reinforcement do not occur when the beam reaches its strength. Domenico and Ricciardi [14] presented an upgraded truss model in which the upper compression strut may show lower inclination compared to the lower compression strut. This is mechanically induced by the increase in shear stress in the upper part of beam close to the crack tip. It was shown that the proposed model accurately estimates the experimental shear strength of RC beams. A number of studies have been performed on the shear behavior of bridge decks affected by ASR [15,16]. Schmidt et al. [15] presented a test method for concrete bridges without disturbing the traffic. Their approach included collecting concrete cylinder samples and unreinforced beams from parts of the slab that were damaged and less damaged by ASR for comparative load testing in the laboratory. Barbosa et al. [16] presented results of tests on 18 RC beams sawn from a flat bridge slab significantly damaged from ASR. The test results showed that the crack distribution and moment capacity of the beams were affected by ASR and the beams failed in flexure under three-point bending but the prestressing effect of the ASR-induced expansion compensates for degraded concrete properties.

ASR, being one of the most prevalent damage mechanisms in RC structures, deserves further investigation with regards to its influence on the shear capacity of RC beams. This study aims to fill this gap of scarce data and analysis on the shear capacity of ASR-damaged beams. First, a comprehensive literature review has been performed to synthesize the available data in terms of the reduction in concrete mechanical properties as a function of ASR expansion. Then, the results of two full-scale shear tests on a RC beam have been presented in terms of maximum shear capacity, crack maps and widths, and strains in the reinforcement. The two shear spans had the same geometry and reinforcement details such that the repeatability of the experiments could be verified. The results from the second shear test in which the beam was pushed to ultimate strength and failure have been used to validate a finite element model (FEM), which was later used to simulate the long-term structural behavior of the beam affected from ASR. The reduction in the mechanical properties of the concrete was estimated using relationships obtained from data synthesized from the literature and then used as inputs to the FEM in a parametric study. The reduction in the shear capacity of the beam was predicted as a function of the concrete ASR expansion. 

## 2. Literature Synthesis

Numerous studies have reported the effect of ASR on the mechanical properties of concrete; particularly, on the compressive and tensile strength, and Young’s modulus. However, the effect on the compressive strength is disputed among researchers [17,18]. Most of the studies reported that the effect of ASR on the tensile strength and Young’s modulus is higher than that on the compressive strength, as further discussed below.

A comprehensive experimental program was performed by the authors of this study on the effect of ASR on the mechanical properties of concrete using accelerated aging [19]. A 0.8% sodium hydroxide by weight of cement was added to concrete and degradation was evaluated over time. Increasing the alkaline dosage is a well-known method in which the long-term behavior of the material is simulated in a short period of time [20,21,22]. Over a 210-day period of accelerated aging, 16% and 21% reduction, respectively, was observed in the split tensile strength and elastic modulus of the concrete. However, the reduction in compressive strength was not significant (about 5%). 

Sanchez et al. [17] prepared 20 concrete mixtures with three target strengths of 25 MPa, 35 MPa, and 45 MPa, and ten different reactive aggregates to study the damage caused by alkali–aggregate reaction (AAR). AAR refers to the general class of reactions between the alkali hydroxides in the concrete pore solution and the mineral phases of the aggregates. ASR is one of the most common types of AAR [23]. Thirty-five cylinder specimens, 100 mm by 200 mm in size, from each mixture were tested at different expansion levels with a maximum expansion of 0.3%. All 20 concrete mixtures had the same amount of cement and aggregate volume for comparison purposes. The cylinders were kept at 38 °C and 100% relative humidity. The highest reduction in elastic modulus was about 67%, and the lowest was about 40% at 0.3% expansion for the 35 MPa mixture. The reduction in tensile strength was between 45% and 80%, depending on the aggregate type at the 0.2% expansion level. The compressive strength was the least affected parameter, with a reduction between 20% and 35% at the 0.2% expansion level. 

Esposito et al. [24] studied the effect of ASR on concrete and developed a correlation between mechanical properties and expansion. Two different concrete mixtures from Dutch and Norwegian aggregates were used. The Norwegian aggregates were expected to have a higher reactivity than Dutch ones. The two concretes were designed with similar aggregate gradation and 28-day compressive strength. The samples from both mixtures were stored at 38 °C and 96% relative humidity. The test data showed a 28-day compressive strength between 60 MPa and 68 MPa. The expansions were measured on 75 × 75 × 280 mm prisms and the compressive testing was performed on 150 mm cubes. The last test was conducted at 365 days of concrete age. The approximate expansion obtained after 365 days was 0.11% and 0.18% for the mixtures using the Dutch and Norwegian aggregates, respectively. A slight increase in the mechanical properties was observed in the first 90 days, and then the degradation began. The variation of the static elastic modulus of the concrete with Dutch aggregates was insignificant (less than 7%), while that with Norwegian aggregates showed a 35% reduction. Both concretes showed an increase in the compressive strength while the split tensile strength exhibited a 23% and 26% decrease for the concretes with Dutch and Norwegian aggregates, respectively. In conclusion, the elastic modulus was found to be the most sensitive parameter to ASR and the second most affected parameter was found to be the split tensile strength. It was concluded that the increase in the concrete strength with increased maturity overshadows a potential decrease due to ASR.

Fan and Hanson [25] performed three-point flexural tests on 152 × 254 × 1524 mm concrete beams affected by ASR over the one-year accelerated aging period. The beams were immersed in an alkali solution that was cyclically heated between 38 °C and room temperature. The alkali solution contained 10 g of sodium hydroxide (NaOH), 14 g of potassium hydroxide (KOH), and 0.1 g of calcium oxide (CaO) per liter of tap water. The expansion in the cylinders was measured using a mechanical dial gage as the change in the distance between the studs placed on the sides of the specimen. It was found that the mechanical properties of nonreactive cylinders do not change significantly over time. However, tests showed about 24%, 38%, and 31% percent reduction in the compressive strength, splitting tensile strength, and dynamic modulus, at an age of 6 months and expansion of 2300 microstrain compared to the 28-day values. It was concluded that, despite visible cracks, the flexural strength of the reactive beams is about the same as those of the nonreactive beams.

Gautam et al. [26] performed an experimental study to identify the degradation in material properties of concrete under multi-axial state of stress. The triaxial expansion of 254 mm cubic specimens, prepared with reactive coarse aggregates, and restrained with rods in uniaxial and biaxial conditions up to 9.6 MPa pressure, was measured. 1.3 kg/m^3^ of NaOH pallets were added to the concrete mixtures to accelerate ASR. To accelerate aging, the specimens were cured in an environmental chamber at 50 °C and 100% relative humidity for 8 months. Compression and splitting tension tests were performed at different ages on 75 mm diameter cores drilled from the specimens. It was observed that the expansion was significantly lower in the restrained directions while the volumetric expansion remained relatively constant. Fifteen percent less volumetric expansion for the biaxially stressed specimens was measured compared to the unstressed specimens. The splitting tensile strength for the unrestrained non-reactive specimens increased by 60% while the reactive concrete showed a 50% reduction. The increase in the strength was attributed to the continuing hydration of the anhydrous cement in the samples. No statistically meaningful change was observed in the compressive strength while an average of 20% reduction was observed in the elastic modulus. The confinement increased the compressive strength while the elastic modulus was similar both in restrained and unrestrained samples.

Ahmed et al. [27] investigated the material properties of concrete subjected to a 12-month accelerated aging regimen. The compressive strength of 100 mm cubes, the split tensile strength of 100 × 100 mm cylinders, and direct tensile strength, according to BS 6319 [28], of dumb-bell briquette-shaped specimens were evaluated. Fused silica (as reactive aggregates) and Portland cement with a high alkali content of 4.9 kg/m^3^ were used in the concrete. Additional Na_2_SO_4_ and K_2_SO_4_ were added to the mixture to increase the alkali content to 7 kg/m^3^. After curing, the specimens were kept in water at 38 °C to accelerate the chemical reactions. Expansions in 100 × 100 × 500 mm prisms and 150 × 300 mm cylinders were measured using a digital Vernier scale. The reduction in the compressive strength was 59% after 12 months at 1.6% expansion. The losses in the split tensile and direct tensile strength were found to be 60% and 80%, respectively.

Sargolzahi et al. [29] evaluated two concrete mixtures, one with Spratt limestone as a reactive aggregate source and the other with Limeridge limestone as a non-reactive aggregate source. Four prisms, 75 × 75 × 285 mm, for expansion measurements and 24 cylinders, 100 × 200 mm, for mechanical testing, were prepared. NaOH pellets were added to the reactive concrete mixture to increase the alkali content to 4.5 kg/m^3^. The specimens were stored at 38 °C in a high humidity environment according to the Canadian CSA A23.2-14A Standard [30]. The compressive strength of the reactive concrete was 14% lower than that of the non-reactive mixture after 23 months of aging and at 0.14% expansion. This reduction was 37% for the static modulus of elasticity under the same conditions.

Saint-Pierre et al. [31] applied accelerated aging on concrete by submerging the specimens made with reactive aggregate in a 1 mol L^−1^ NaOH solution at 38 °C for 3.5 months. The alkali content (Na_2_O equivalent) of the reactive concrete was 5.25 kg/m^3^. Ten prisms, 75 × 75 × 300 mm, with reference studs for expansion measurement and 40 cylinders, 100 × 200 mm, for mechanical tests were prepared. The maximum expansion in the reactive and non-reactive concretes was measured as 0.19% and 0.02%, respectively. While the difference between compressive strength for the reactive and non-reactive mixtures was insignificant, a 13% reduction in the elastic modulus was observed.

Smaoui et al. [32] evaluated the mechanical and durability properties of concrete affected by accelerated ASR. Low-alkali and high-alkali content mixtures were prepared with 0.6% and 1.25% Na_2_O by mass of cement, respectively. The high-alkali mixture was prepared by dissolving NaOH pellets in the mixing water. Non-reactive coarse limestone and fine granitic aggregates were used for both concretes with a water-to-cement (w/c) ratio of 0.41. The specimens were kept at 23 °C and 100% relative humidity until testing. Prisms 50 × 75 × 350 mm in size and 100 × 200 mm cylinders were tested at various ages up to 180 days. The results revealed the difference between the properties of concrete samples made from high-alkali and low-alkali mixtures. At an age of six months, the high-alkali concrete specimens showed 12% lower compressive strength, 7% lower modulus of elasticity, 15% lower direct tensile strength, 14% lower split tensile strength, and 3% lower modulus of rupture compared with the low-alkali concrete specimens.

Multon et al. [33] performed material testing as a part of an investigation on the effect of moisture gradient on ASR. Potassium hydroxide (KOH) was dissolved in the mixing water to increase the Na_2_O equivalent content of the mixture to 1.25%. Cylinders with measurements of 160 × 320 mm were tested for mechanical properties. The samples were kept at 38 °C for 12 months in sealed conditions. The elastic modulus of the reactive concrete was 20% lower at 12 months compared to the 28-day value and 27% lower compared to the non-reactive concrete. The tensile strength was 3% higher after 12 months compared to the 28-day value but 9% lower compared to the non-reactive concrete of the same age. The reactive concrete also had a 10% increase in compressive strength after 12 months but the difference with the non-reactive concrete was indistinguishable.

Giannini and Folliard [34] used El Paso, Texas sand and New Mexico gravel to prepare two reactive concretes. Cylinders with measurements of 100 × 200 mm were prepared for compressive testing. Type I cement was used and NaOH was added to the mixture to increase the alkaline content to a Na_2_O equivalent of 5.25 kg/m^3^. The cylinders were initially wrapped with plastic to prevent moisture loss and then stored at 38 °C and 95% relative humidity. In the El Paso sand concrete, the compressive strength and elastic modulus showed a 13% and 45% reduction, respectively, after one year of aging and 0.6% expansion compared to their 28-day values. The New Mexico gravel concrete showed a 10% increase and a 19% reduction in the compressive strength and elastic modulus, respectively, after one year of aging and 0.85% expansion.

**Figure 1:**
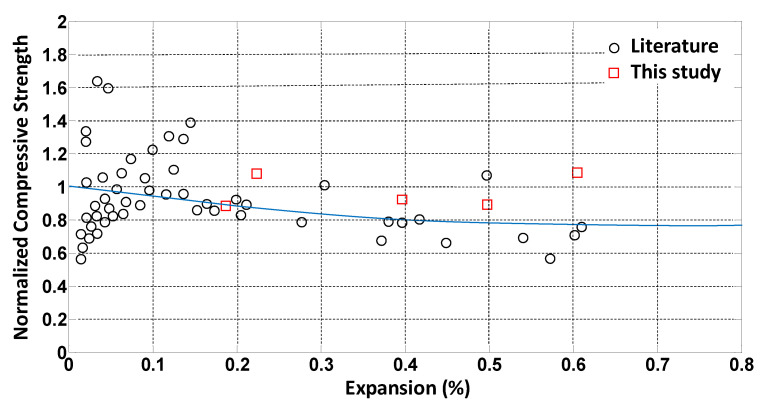
Normalized compressive strength versus ASR expansion.

**Figure 2:**
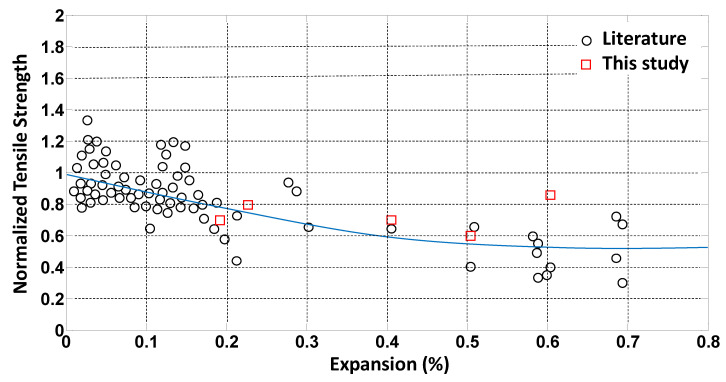
Normalized tensile strength versus ASR expansion.

**Figure 3:**
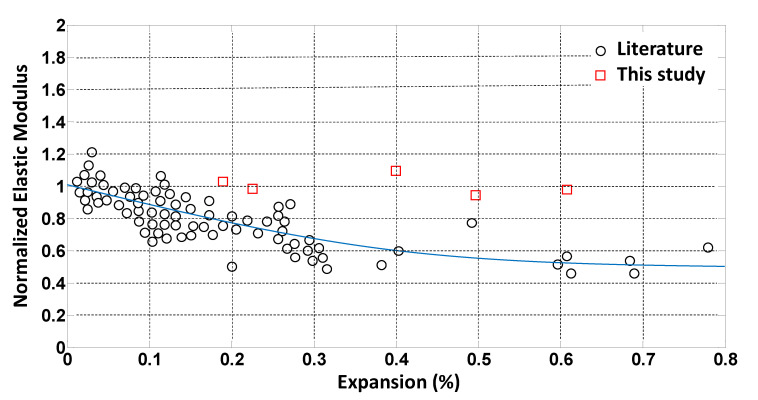
Normalized modulus of elasticity versus ASR expansion.

More than 50 data points for elastic modulus, and compressive and tensile strength were extracted from the literature, as listed in Table 1 and plotted in Figure 1, Figure 2 and Figure 3. The data points indicated as “current study” in Table 1 were obtained by the authors, as presented in [24]. The data points were normalized with respect to the 28-day strength value of each mixture. Exponential trendlines were obtained for each set of data and presented in the figures. The equations for these trendlines are summarized in Table 2. These equations are used to estimate material degradation as inputs to the parametric study performed using the FEM. 

## 3. Experimental Program

The experimental program and the results are described in detail in a recent study by the authors [46]. Since the validation of the computational model in this study is based on the results of these experiments, a brief summary of the pertinent parts of the experimental program and results are presented in this section.

Concrete with a w/c ratio of 0.52, a slump of 190 mm and an average 28-day strength of 27.5 MPa was designed as shown in Table 3.

To obtain the mechanical properties of the concrete, standard material tests were performed at 28 days and 210 days after casting, the latter of which being when the beam was tested. Table 4 shows the results obtained from these tests. As presented in Table 4, the split tensile strength of concrete decreased as a result of early-stage ASR degradation. The scatter of the test results in Table 4 can be found in the figures of [6].

**Figure 4:**
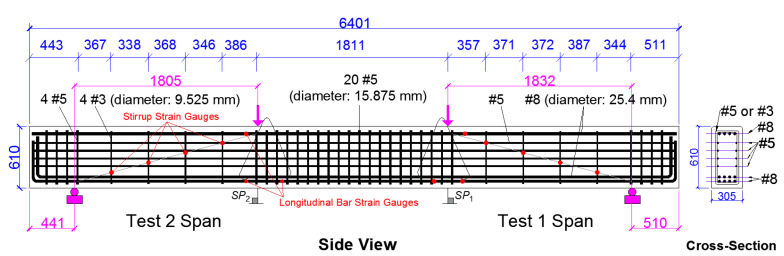
As built dimensions of the full-scale beam, loading and support locations and instrumentation (all dimensions are in mm).

A full-scale RC beam, 6.4 m in length with a rectangular cross-section 0.6 m deep and 0.3 m wide, was fabricated. The volumetric ratio of the gross longitudinal reinforcement was constant at 4.2% over the length of the beam, while a light transverse reinforcement with a volumetric ratio of 0.13% was provided in the two shear spans with a span-to-depth ratio of about 2.9, as shown in Figure 4. To calculate this ratio, the span length was measured between the center of the loading plate and the center of the support plate. At the center of the beam and above the supports at the ends, a heavy shear reinforcement with a volumetric ratio of 1.3% was used to prevent failure in these regions. Figure 4 also shows the locations of supports and loading points during the first and second tests as well as the string potentiometers, *SP*_i_, for deflection measurements under the loading points. Additionally, the strain gauges installed on the stirrups and longitudinal reinforcement are shown in Figure 4. The actuator load and displacement were also, respectively, obtained from the actuator load cell and actuator linear resistance transducer (LRT). All the sensors, including load cells, LRT, and string and linear potentiometers, were calibrated and verified prior to testing. No calibration is required for the strain gauge sensors and these strain gauges were installed on the reinforcement by grinding and polishing a smooth surface on the steel and then, attaching and sealing the sensors.

**Figure 5:**
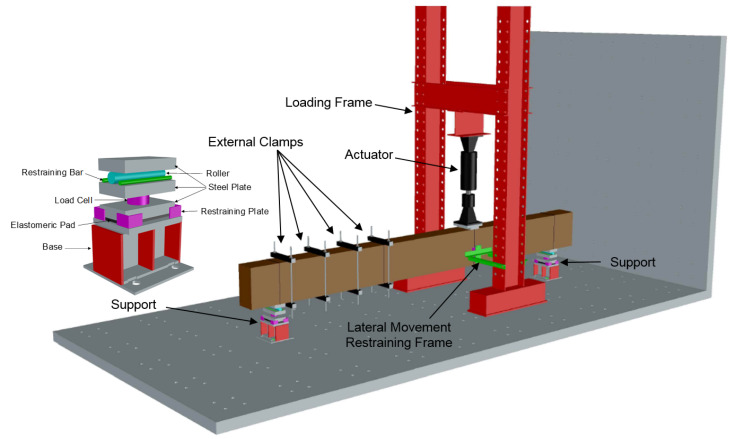
Test setup.

The test setup consisted of the components shown in Figure 5. The supports at the two ends allowed beam displacement in the longitudinal direction and rotation about the strong axis. A loading frame and a servo-hydraulic actuator with 670 kN capacity were used to apply load to the shear span. Restraints were placed on either side of the beam to restrict the movement of the beam to the vertical direction within the test plane. External clamps were installed in the shear span that was not being tested to protect it from cracking and damage, and to allow for a second test on the same beam. A total of 890 kN post-tensioning force was applied through these clamps, which were pre-analyzed to be sufficient to prevent cracking in this region. The clamps were also used during the second test, but this time on the already tested and cracked span, so that a complete failure in the already tested span could be prevented and the shear deformation could be focused into the span being tested.

**Figure 6:**
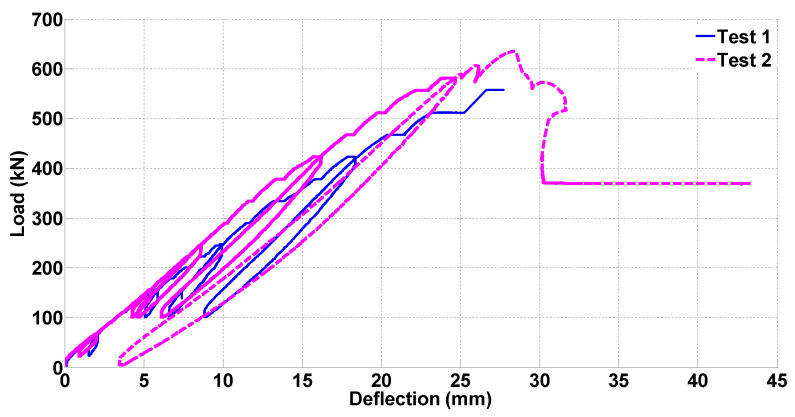
Load–deflection diagrams for the shear tests. The deflection was measured under the loading point.

The tests were conducted under quasi-static loading. As described in [46] in detail, the loading protocol included multiple steps of loading, pauses to conduct crack measurements, and unloading. The loading protocol consisted of load control and displacement control segments. Only during the displacement control segment that was performed at the final stage of the second test to push the beam to failure, was there a direct relation between the load rate and the beam deflection. In the other segments, the deflection of the beam was a function of the tangent stiffness of the beam at that stage of the loading and the load increment or decrement. Load–deflection diagrams for the first and the second shear tests performed on the two ends of the beam are presented in Figure 6. During the first shear test, the beam was not loaded to complete failure and the test was terminated at 556 kN load to prevent damage to the other shear span. In the second test, the loading was continued up to 578 kN in load-control mode and then switched to displacement control and the test was continued up to complete failure. The maximum capacity of the beam was obtained as 635 kN in the second test and the residual shear strength of the beam after failure was found to be 369 kN. Figure 6 shows that in the second shear test, the beam retains a slightly higher stiffness past 8 mm deflection. This slight difference is attributed to the uncertain clamping force applied to the other span during each test, and the influence of the cracks caused by the first test on the second test.

**Figure 7:**
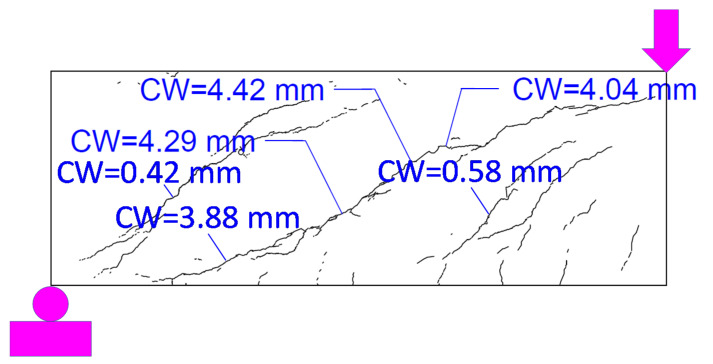
Crack maps and width at the peak load of 635 kN.

Although both tests resulted in a shear failure, since during the second test the beam was pushed to the complete failure, for brevity, the results of the second shear test are presented in the following. Figure 7 presents the crack maps and major crack widths at the peak load of 635 kN. The widths of the cracks were larger close to the middle of the shear span. Figure 7 only presents the crack pattern and crack widths at select locations. The crack width is not presented to scale. Crack widths were manually measured at select locations, as shown in the figure, that have been visually identified to be the largest crack opening.

**Figure 8:**
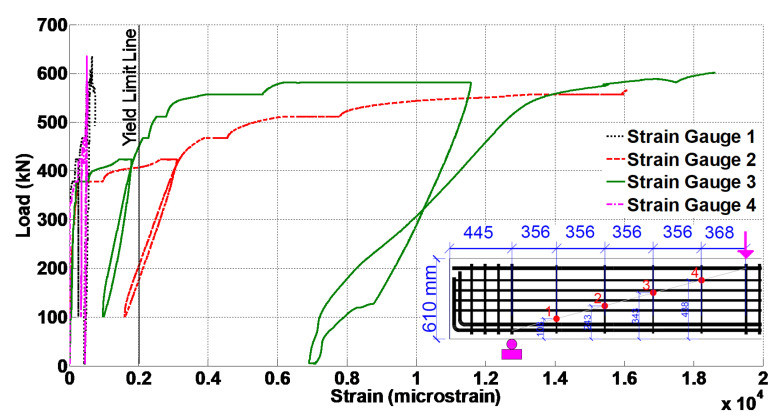
Load versus stirrup strains.

Figure 8 presents the results of stirrups strain versus load. The strain gauges #2 and #3, which were installed on the middle stirrups of the span, experienced significantly higher strains compared to the other two strain gauges. This is consistent with the results presented in Figure 7 for the crack widths in the middle of the span.

## 4. Finite Element Modeling and Analysis

### 4.1. Material Models

As mentioned earlier, a FEM is created for the shear failure of the RC beam using ATENA 3D [52]. The selected constitutive model for concrete, CC3DNonLinCementitious2, is based on fracture in tension and plasticity in compression. The parameters used for this model are summarized in Table 5. Some parameters such as elastic modulus, Poisson’s ratio, tensile strength, and compressive strength were obtained from material tests, and the remaining parameters, including specific energy, tension stiffening, and crack shear stiffness factor, were determined through the model tuning and validation using the data from the beam experiment. The definitions of the parameters given in Table 5 for the CC3DNonLinCementitious2 concrete model are presented in detail in ATENA program documentation [52]. The value of 1 for the fixed crack model coefficient in Table 5 indicates that this is the active mode for concrete cracking. In this model, the crack distribution is uniform within the material volume and cracking occurs when the principal stress exceeds the tensile strength of concrete. In the fixed crack model, the direction of crack during loading remains fixed, as given by the principal stress direction at the moment of crack initiation. The crack width is calculated based on the crack opening strain over the crack band in the finite elements representing the concrete.

A bilinear model with strain hardening, CCReinforcement, was used to model the steel reinforcing bars. The input parameters were derived from the uniaxial rebar testing for different steel reinforcements in the beams as presented in Table 6.

### 4.2. Geometry

**Figure 9:**
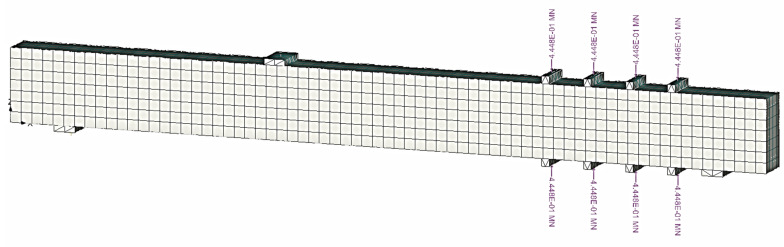
Finite element mesh and post-tensioning force.

**Figure 10:**
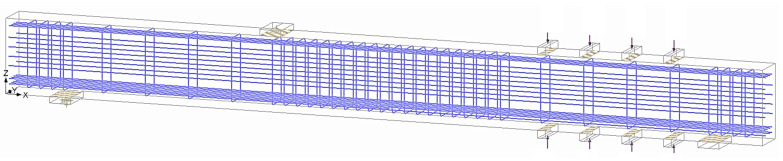
Flexure and shear reinforcement in the FEM.

The geometry of the model is based on the dimensions of the beam presented earlier. The model is composed of three parts: the concrete beam, reinforcement, and the steel plates for the supports, loading plate and the clamps. The concrete was meshed with iso-parametric 3D linear brick elements (see Figure 9). To model the post-tensioning force of the clamps, a discrete rigid object with the same contact area of the clamps was created on the top and bottom of the beam, and a compression force, equal in magnitude to that was used in the experiments, was applied, as shown in Figure 9. The embedded discrete reinforcement (shown in Figure 10) had displacement compatibility with the surrounding concrete. Table 7 presents the mesh information of the FE model for concrete, steel (supports and loading points), and rebar components.

### 4.3. Loading and Boundary Conditions

The load was simulated by applying a 25.4 mm prescribed deformation, for a complete analysis, at increments of 0.01 mm displacement in the negative Z, vertical, direction. The supports were free to rotate about the transverse axis of the beam and they were restricted to move in the vertical direction. Surface springs were placed under the supports to represent the behavior of elastomeric pad of the supports shown in Figure 5, and a 890 kN load was applied to simulate the effect of the clamps in the farther shear span of the beam.

### 4.4. Solution Method

The Newton–Raphson method with the line search optimization approach and tangent type stiffness was used for solving the governing equations. The line search strategy adjusts the displacement increments to optimize the required work and reduces the out-of-balance forces. Table 8 presents the iteration limit and different error tolerances for the solution method along with the parameters of the line search approach.

### 4.5. Model Validation

**Figure 11:**
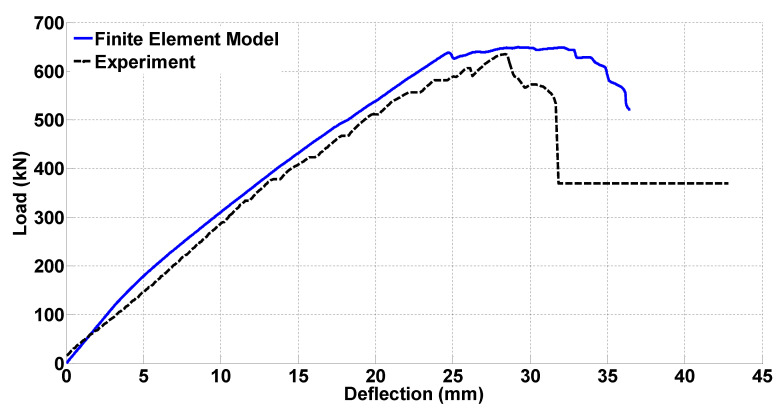
Load versus deflection diagrams of the experiment and the FEM.

The FEM is validated using the experimental data from the second test. Like the second shear test, the first test also resulted in shear failure but the shear failure of the second test included the ultimate shear load capacity and the load drop since the test continued to capture the entire failure. The primary step taken for validation is to compare the load versus deflection diagrams, which is shown in Figure 11. Despite some differences in the stiffness and the ultimate strength, an acceptable agreement was obtained.

**Figure 12:**
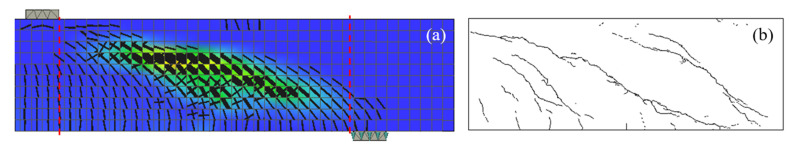
Crack patterns of the (**a**) FEM and the (**b**) experiment at peak load.

For further evaluation of the FEM, the crack patterns corresponding to the peak load are compared, as shown in Figure 12. Similar to Figure 7, Figure 12b presents the crack pattern, and the concentration and widths of the cracks are not presented to scale. Overall, the crack patterns show a good correlation between the FEM and the experiment. In both patterns, the major shear crack was located diagonally between the loading point and the support. The maximum crack width at peak load was obtained as 4.1 mm from the FEM and 4.4 mm from the experiment.

**Figure 13:**
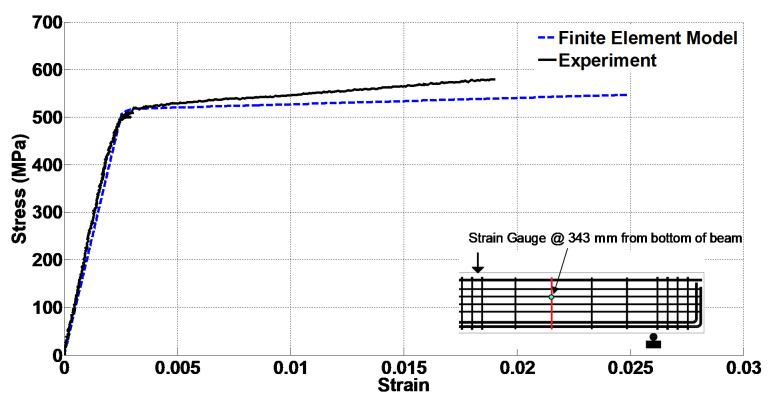
Comparison of the experimental and numerical stress–strain diagrams in the stirrup close to the middle of the span.

**Figure 14:**
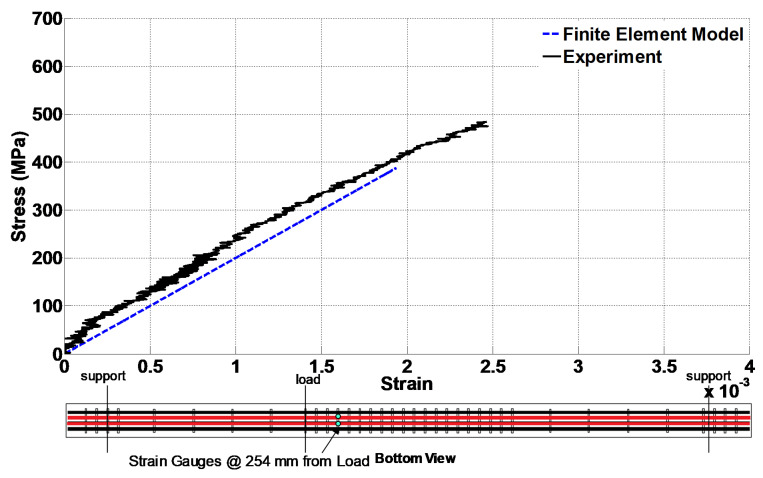
Comparison of the experimental and numerical stress–strain diagrams of the bottom longitudinal rebar close to the loading point.

The FEM was also validated by comparing the reinforcement strains. From four #3 stirrups, the ones close to the middle of the span showed a significant strain. Therefore, in Figure 13, the stress versus strain diagrams of the stirrup close to the middle of the shear span are compared. The experiment stirrup strain at the peak load was 0.019, while the strain from the FEM was about 0.025. Figure 14 presents the stress versus strain diagrams of the bottom longitudinal rebar. It is noted that the stress values for the experimental data were obtained from the material tests of the rebar and using the rebar strains recorded from the strain gage. Additionally, the diagrams in Figure 13 and Figure 14 are sketched up to the strain corresponding to the peak load. These figures indicate acceptable agreement between the results of the experiment and FEM. Additionally, the bottom longitudinal steel reinforcement showed 0.0019 strain at the peak load during the experiment, while this value was 0.0024 in the FEM.

### 4.6. Parametric Study

In this section, the validated FEM is used to evaluate the influence of ASR on the shear performance of the beam. This investigation is based on the degradation in the concrete mechanical properties due to ASR. The mechanical properties affected by ASR are chosen as the concrete compressive and split tensile strength, and elastic modulus. The equations extracted from the literature, as presented in Table 2, were used to estimate the degradation in these properties. In addition, the reduction in the fracture energy, which is an important property of the concrete constitutive model used in the FEM, has been also calculated using

(1) $$G_{F}=73\;f_{cm}^{0.18}\;\;\;\;\;\;\;$$

where *G_F_* is the fracture energy (N/m) and *f_cm_* is the mean compressive strength (MPa) [53]. Three cases are defined based on ASR expansion levels of 0.2%, 0.4%, and 0.6%. Accordingly, the normalized values of the concrete properties are calculated for different expansions, as presented in Table 9.

**Figure 15:**
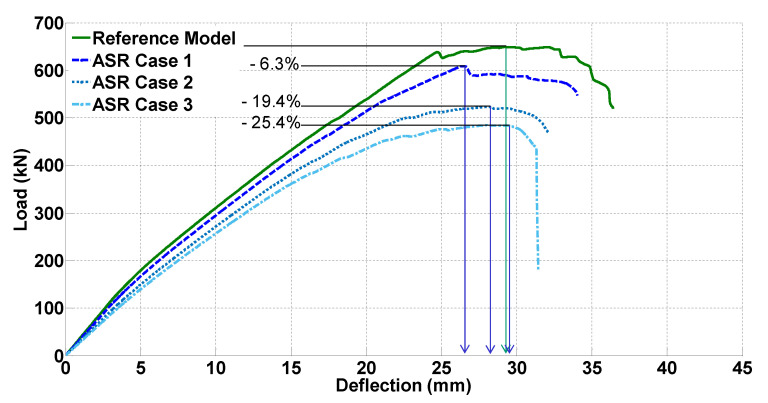
Load versus deflection diagram for different levels of ASR degradation.

Figure 15 presents the effect of ASR on the shear performance of the RC beam in terms of load versus deflection. The reference model and the models representing concrete degradation due to ASR are shown. According to Figure 15, ASR Cases 1 to 3 cause a reduction in the shear strength by 6.3%, 19.4%, and 25.4%, respectively. It is noted that these shear strength reductions are merely the result of degradation of concrete properties obtained at different ASR expansions. The potential self-stressing of concrete as a result of ASR expansion in the presence of internal reinforcement, which may act to recover the strength reduction, is not considered. Therefore, these shear strength reductions for different ASR cases are conservative estimates.

**Figure 16:**
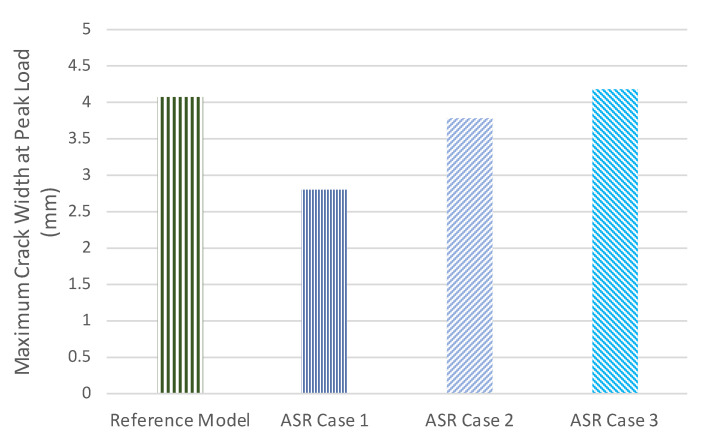
Maximum crack width at peak load for different cases of ASR.

Figure 16 presents the maximum crack width at peak load for different levels of ASR degradation. According to this figure, the maximum crack width at peak load for ASR Cases 1 and 2 is less than that of the reference model. For Case 3, the maximum crack width at peak load is slightly higher than the crack width in the reference model. This result is attributed to the deflection corresponding to the peak load for each model. For example, as shown in Figure 15, compared to the reference, ASR Case 1 has smaller deflection at peak load. For higher ASR expansions, the deflection at peak load increases, which results in larger maximum crack widths corresponding to the peak load.

Table 10 presents the maximum strain values corresponding to peak load at the bottom and top longitudinal rebar as well as the four stirrups in the shear span for different cases of ASR. The stirrup strain gauge numbers in Table 10 refer to those in Figure 4. A full bond between the reinforcement and the concrete was assumed in the FEM. The results from Table 10 indicate the relation between the maximum reinforcement strains at peak load and different levels of concrete degradation. Accordingly, a low level of ASR degradation reduces the stirrup strains. At higher levels of ASR degradation, the stirrup strains increase and, in most cases, exceed the strain values from the reference model. This is related to the deflection at the peak load. As shown in Figure 15, the deflection at peak load for low and high levels of ASR damage is smaller and higher, respectively, than that of the reference model. ASR Case 1 applies a lower degradation in concrete mechanical properties than that of other cases which do not significantly change the beam shear performance. This results in a similar but smaller stiffness in the load–deflection diagram and consequently slightly smaller peak load and the corresponding deflection. With an increase in the ASR degradation of concrete mechanical properties, the significant reduction in the maximum load and the stiffness affects the shear performance of the beam. This reduction results in an increase in the deflection at lower peak loads. Table 10 also shows that the strain of top longitudinal reinforcement remains almost unchanged for different levels of ASR. However, for the bottom longitudinal reinforcement, as the ASR becomes worse, the strain value drops. Unlike the stirrup strains, which depend on the deflection at peak load, the strain of the bottom longitudinal reinforcement at peak load is more dependent on the value of the load. As the load capacity decreases due to ASR, the strain of the bottom longitudinal rebar at the peak load drops. The reason for this is that in shear failure, the plastic shear deformation develops disproportional to the load and determines the strain of the resisting stirrups. Meanwhile, due to shear failure occurring prior to any flexural yielding, the bottom longitudinal reinforcement strains are almost proportional to the load. Table 10 shows this relation between the strain values in the reinforcement and the ASR level. This table also shows that, unlike the longitudinal steel reinforcement, all the indicated stirrups in all the FEM yield at the peak load. This confirms that, as desired, the nonlinear shear behavior governed the beam response while the beam stayed in the linear range until the peak load was achieved.

**Figure 17:**
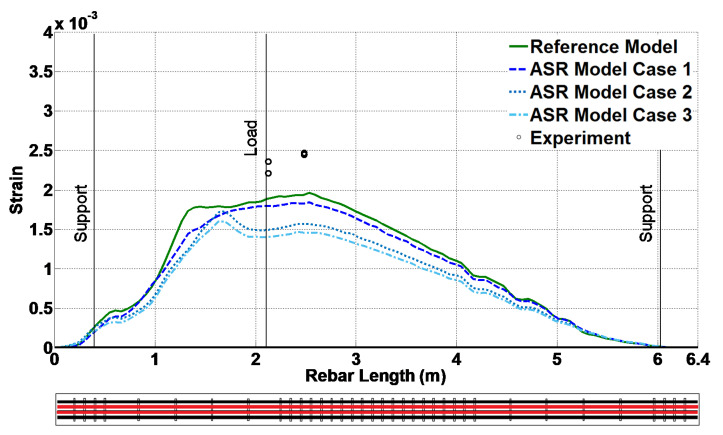
Strain along the length of the bottom longitudinal rebar at peak load.

**Figure 18:**
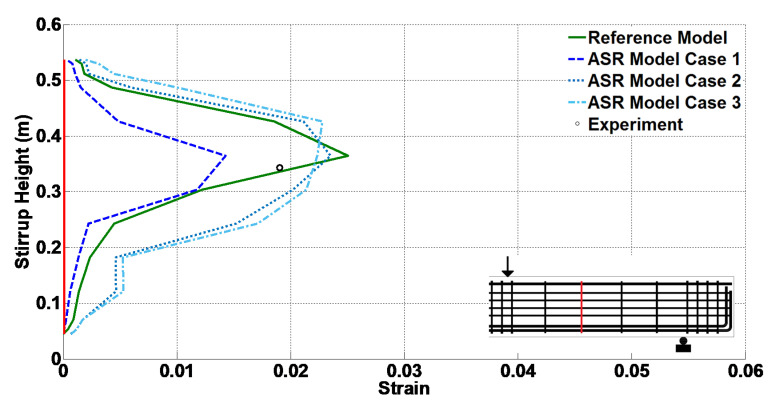
Strain along the height of #3 stirrup at peak load.

Additionally, for different ASR cases, Figure 17 and Figure 18 illustrate the variation of strain at peak load along the length of the bottom longitudinal rebar and the height of the middle stirrup, respectively. The experimental results are also shown using circular markers at the strain gauge locations. It is noted that the peak values in these plots correspond to the results of Table 10.

## 5. Conclusions

As a part of this study, a large-scale beam was constructed and tested to study its shear performance. The beam was designed to fail in shear prior to any flexural yielding. Two shear tests were conducted by applying a load to each shear span of the beam, with span-to-depth ratios of about 2.9 at each end. The shear tests were conducted under quasi-static loading. The acquired data were processed, and the experimental results, including shear load versus deflection diagram, reinforcement strains, crack maps and maximum crack width at peak load were used to validate a detailed FEM of the beam. The validated FEM was in turn used to study the effect of different levels of ASR degradation corresponding to 0.2%, 0.4%, and 0.6% of the concrete expansion. The reduced concrete compressive strength, tensile strength, modulus of elasticity, and fracture energy were calculated based on ASR expansion and used in the FEM. The following conclusions are made:

- The shear tests indicated that the nonlinear shear behavior governed the response of the beams with span to depth ratios of 2.9, a shear reinforcement ratio of 0.13%, and a longitudinal reinforcement ratio of 4.2%. This was confirmed based on the data from the strain gauges installed on the shear and longitudinal reinforcement.
- The major diagonal shear cracks were from the top corner loading point towards the bottom corner support. Some secondary diagonal shear cracks also occurred between the top corner loading point and the middle of the span at the bottom and between the bottom corner support and the middle of the span at the top.
- The major shear cracks were more concentrated in the middle of shear span than towards the ends. Similarly, the stirrups close to the middle of shear span yielded while the stirrups close to the loading point and support remained elastic throughout the test.
- According to the parametric study using the validated FEM, 0.2%, 0.4%, and 0.6% of ASR expansion induced 6.3%, 19.4%, and 25.4% reduction in the shear capacity of the beams.
- For ASR degradation less than 0.4% expansion, the maximum crack width at peak load decreased compared to the reference model but as the ASR degradation progressed, the maximum crack width at peak load increased. For the highest level of ASR degradation with 0.6% expansion, the maximum crack width at peak load slightly exceeded that of the reference model. This happened due to the significant reduction in the beam stiffness, which caused higher deflection at a much lower peak load.
- For ASR degradation less than 0.2% expansion, the stirrup strain at peak load is reduced compared to the no ASR case. However, for higher levels of ASR with 0.4% and 0.6% expansion, the strains of the stirrups exceeded the strains in the reference model. This is because the stirrup strain is related to the deflection. The deflection at peak load is smaller at the early stages of ASR but then it increases as the ASR further develops due to further reduction in the material properties. Early-stage ASR results in a slightly lower peak load and corresponding deflection. Higher ASR degradation considerably affects the beam shear performance and reduces the peak load and stiffness. This causes more deflection in the beam and higher stirrups strain.
- For different levels of ASR degradation, no significant variation in the top longitudinal reinforcement strain at peak load was observed. On the other hand, as the level of ASR degradation increased, the strain of the bottom longitudinal reinforcement at peak load decreased. Unlike the stirrup strains, which were correlated to the deflection at peak load, the strain of the bottom longitudinal reinforcement was correlated to the peak load.

The computational models in this study were developed to capture the degradation of beam shear strength as a result of degradation of the concrete mechanical properties from ASR. The relation between the concrete degradation and ASR expansion was derived from the literature and implemented in the models but the models did not consider the influence of the self-prestressing effect as a result of concrete expansion. Therefore, further research is needed to quantify the self-prestressing effect as a function of the ASR expansion, reinforcement size and ratio, and concrete properties, and if significant implement this effect in modeling the behavior of reinforced concrete members affected from ASR.

## Figures and Tables

**Table 1:** List of the studies on ASR-affected concrete properties.

Reference	Reactive Aggregate Type	Cement Content (kg/m^3^)	w/c ^1^ Ratio	Alkaline Dosage (%) ^2^	Maximum Expansion (%)
Current study	El Paso Sand	291	0.52	1.25	0.18
Attar et al. [19]	El Paso sand	427	0.33	1.25	0.10
Smaoui et al. [35]	Texas sand	420	0.4	1.25	0.39
Smaoui et al. [35]	Québec City limestone	420	0.4	1.25	0.17
Esposito et al. [24]	Quartzite, quartz, chert, volcanic rock	380	0.45	1.17	0.11
Esposito et al. [24]	Quartz, quartzite, gneiss, metarhyolite	380	0.45	1.17	0.18
Sargolzehi et al. [29]	Spratt limestone	345	0.5	1.25	0.08
Fan et al. [36]	Gold Hill coarse aggregate	403	0.47	- ^3^	0.17 ^4^
Saint-Pierre et al. [31]	Spratt limestone	350	0.5	5.25 ^5^	0.2
Giaccio et al. [37]	Crushed quartzitic sandstone	380	0.42	4.0 ^5^	0.22
Giaccio et al. [38]	Granitic stone with feldspars, quartz, etc.	420	0.42	1.24	0.28
Multon et al. [33]	Limestone	410	0.5	1.25	0.26
Giannini et al. [34]	Dolomite, carbonate, siliceous volcanics	420	0.42	1.25	0.14
Giannini et al. [34]	El Paso sand	_	_	_	0.42
Swamy and Al-Asali [39]	Amorphous fused silica sand	520	0.44	1.00	0.62
Larive [40]	Tournaisis limestone	410	0.44	1.25	0.21
Monette [41]	Siliceous limestone	423	0.61	1.25	0.35
Ahmed et al. [27]	Thames Valley sand (50% chert) and fused silica	400	0.5	1.75	0.15
Multon [42]	Calcareous stones with silica	410	0.5	1.25	0.1
Ben Haha [43]	Chlorite interleaved with quartz and feldspar	_	0.46	0.4	0.16
Lindgård [44]	Ottersbo cataclasite with crypto and quartz	550	0.3	0.67	0.08
Lindgård [44]	Ottersbo cataclasite with crypto and quartz	400	0.45	0.93	0.23
Lindgård [44]	Ottersbo cataclasite with crypto and quartz	315	0.6	1.17	0.28
Sanchez et al. [45]	Volcanics and chert sand	314	0.61	1.25	0.3
Sanchez et al. [45]	Volcanics and chert coarse aggregate	314	0.61	1.25	0.2

^1^ Water-to-cement ratio; ^2^ by weight of cement; ^3^ the specimens were submerged in 0.5 N concentration alkali solution for 6 months; ^4^ maximum expansion on reinforced beam; ^5^ the total alkali content in the mixture (kg/m^3^).

**Table 2:** Normalized equations for estimating ASR damage as a function of expansion.

Mechanical Property, y	Property, y as a Function of Expansion Percentage, x
Elastic Modulus	y = 1.0189 × 10^−1.224x^
Tensile Strength	y = 0.9830 × 10^−1.068x^
Compressive Strength	y = 1.0111 × 10^−0.482x^

**Table 3:** Concrete mixture proportions.

Material	Amount
Mixing Water, kg/m^3^	154
Type II Low Alkali Portland Cement, kg/m^3^	291
19 mm Coarse Aggregate, kg/m^3^	1038
9.5 mm Coarse Aggregate, kg/m^3^	208
Reactive Sand from El Paso, Texas, kg/m^3^	727
Pozzolith 8 ^1^, g/m^3^	1187
Glenium 3400 NV ^1^, g/m^3^	1557

^1^ Pozzolith 8 and Glenium 3400 NV are water-reducing admixtures produced by BASF Corporation (Ludwigshafen, Germany) [47].

**Table 4:** Mechanical properties of concrete.

Property	Age		No. of Tests at Each Age	Standard
	28 Days	210 days		
Compressive Strength (MPa)	38.5	40.0	3	ASTM C39 [48]
Split Tensile Strength (MPa)	4.6	3.5	3	ASTM C496 [49]
Elastic Modulus (GPa)	17.7	19.0	3	ASTM C469 [50]
Modulus of Rupture (MPa)	4.7 ^1^	4.8	2	ASTM C78 [51]

^1^ Testing performed at 3 months of age.

**Table 5:** Parameters of the concrete model.

Parameter	Symbol	Unit	Value
Elastic Modulus	E	MPa	1.896 × 10^4^
Poisson’s Ratio	μ	-	0.20
Tensile Strength	f_t_	MPa	4.00
Compressive Strength	f_c_	MPa	−40.00
Specific Fracture Energy	G_F_	MN/m	2.000 × 10^−4^
Tension Stiffening	c_ts_	-	0.04
Critical Compressive Displacement	W_d_	m	−1.050 × 10^−3^
Plastic Strain at Compressive Strength	ε_cp_	-	−3.200 × 10^−3^
Reduction of Compressive Strength Due to Cracks	r_c,lim_	-	0.80
Crack Shear Stiffness Factor	S_F_	-	20.00
Aggregate Size	-	m	0.019
Failure Surface Eccentricity	-	-	0.52
Multiplier for The Plastic Flow Direction	β	-	0.00
Specific Material Weight	ρ	MN/m^3^	2.30 × 10^−2^
Fixed Crack Model Coefficient	-	-	1.00

**Table 6:** Parameters of the reinforcement model.

Material Characteristic	Unit	#5 Stirrup	#5 Longitudinal Rebar	#8 Longitudinal Rebar	#3 Stirrup
Elastic Modulus, E	MPa	200,000	200,000	200,000	200,000
Yield Strength, σ_y_	MPa	483	500	490	517
Maximum Stress, σ_t_	MPa	680	676	672	648
Maximum Strain, ε_lim_	-	0.2	0.2	0.3	0.1
Specific Material Weight, ρ	MN/m^3^	0.0785	0.0785	0.0785	0.0785

**Table 7:** Mesh information of the FEM.

Model Component	Element Shape	Element Size	Element Type	No. of Elements
Concrete	Brick	60 mm	Linear	5300
Steel	Tetrahedral	12 mm	Linear	50,436
Rebar	Line	60 mm	2-D truss	3290

**Table 8:** Solution parameters.

Title	Parameter	Value
General	Iteration limit per analysis step	500
	Displacement error tolerance	0.010000
	Residual error tolerance	0.010000
	Absolute residual error tolerance	0.010000
	Energy error tolerance	0.000100
Line Search	Unbalanced energy limit	0.800
	Limit of line search iterations	2
	Line search limit—min.	0.010
	Line search limit—max.	1.000

**Table 9:** Different cases of ASR and corresponding percentage remained in concrete mechanical properties.

Properties	Case		
	1 0.2% Expansion	2 0.4% Expansion	3 0.6% Expansion
Compressive Strength	92% (36.8 MPa)	83% (33.2 MPa)	76% (30.4 MPa)
Elastic Modulus	80% (15.2 GPa)	62% (11.8 GPa)	49% (9.3 GPa)
Tensile Strength	79% (3.2 MPa)	64% (2.6 MPa)	52% (2.1 MPa)
Fracture Energy	98.5% (0.197 kN/m)	96.7% (0.193 kN/m)	95.1% (0.190 kN/m)

**Table 10:** Maximum reinforcement strain at peak load for different levels of ASR degradation (refer to Figure 4 for numbering of stirrup strain locations).

FE Model	Maximum Strain (×10^−3^)					
	Bottom Longitudinal Rebar	Top Longitudinal Rebar	Stirrup No. 1	Stirrup No. 2	Stirrup No. 3	Stirrup No. 4
Reference	1.96	−1.34	6.56	17.30	25.14	5.38
Case 1	1.85	−1.35	4.50	15.25	14.43	2.96
Case 2	1.73	−1.32	9.94	22.41	23.62	10.10
Case 3	1.60	−1.34	9.82	22.43	22.86	9.37

## Data Availability

The data presented in this study are available upon reasonable request from the corresponding author.
